# Active trachoma and associated factors in children aged 1 to 9 years living in Sasiga rural districts, East Wallaga Zone, Ethiopia, in 2022: a community- based cross-sectional study

**DOI:** 10.1186/s12886-024-03558-8

**Published:** 2024-07-12

**Authors:** Matiyos Lema, Melese Chego Cheme, Adisu Tafari Shama, Dufera Rikitu Terefa, Edosa Tesfaye Geta, Zelalem Desalegn

**Affiliations:** Department of public health, Institute of health sciences, Wallaga University, Nekemte, Ethiopia

**Keywords:** Active trachoma, 1–9-year-old children, Sasiga, Rural district

## Abstract

**Background:**

Trachoma, caused by the bacteria Chlamydia trachomatous, is a contagious eye condition that frequently affects children and quickly spreads from child to child and from child to caregiver. The study aimed to assess the distribution of active trachoma and its associated risk factors among children 1–9 years aged in Households of Sasiga Rural District, Western Ethiopia, in 2022.

**Methods:**

A community-based cross-sectional study was conducted among 577 randomly selected children from March to May 2022. A multistage sampling technique was used. Data was collected through an interviewer-based questionnaire, physical observation, and clinical eye examinations. Epi Data 3.1 was used for data entry. The data were analyzed with SPSS version 24. Bivariate and multivariate binary logistic regressions were performed. In multivariable logistic regression analysis, the adjusted odds ratio with a 95% confidence interval was used to identify factors associated with active trachoma. A *P*-value of 0.05 was considered statistically significant.

**Results:**

The distribution of Active Trachoma among children 1–9 years aged in Households of the study area was 9.5%(95%CI:7.11,11.89). Being from a low-income household with a monthly income of less than 1500 ETB [AOR = 3.49, 95% CI: 1.39, 8.75], Households where the nearest water supply is more than 30 min away [AOR = 8.34, 95%CI: 1.89, 36.73], households with only one room [AOR = 2.98, 95%CI: 1.027, 8.68], and presence of feces in the compound of the households [AOR = 3.08, 95%CI: 1.41, 6.75] were associated with active trachoma in 1–9 years aged children living in the study setting.

**Conclusion:**

The distribution of Active Trachoma among children 1–9 years aged in Households of the study area was found to be high. Monthly income, the time it took to get water for home use, and the presence of feces in household compounds were all linked to active trachoma in children living in the study area. As a result, continuous sanitary education on trachoma transmission and prevention should be strengthened in the district.

## Introduction

Trachoma is a neglected eye disease that is a leading cause of preventable corneal blindness. Early childhood infections can lead to scarring and visual impairment. Infection is spread through ocular and nasal secretions that are passed from person to person on fingers, fomites (such as bedding and washcloths), and eye-seeking flies (particularly Musca sorbens). It is linked to “active trachoma,” a type of inflammatory conjunctivitis. These infections primarily affect children, peaking between the ages of 1 and 5, and then declining [1].

Trachoma has been eradicated in high-income countries due to improvements in hygiene and sanitation, but the disease remains a problem in developing countries [2]. There are 27.8 million cases of active trachoma (68.5% of all cases) and 3.8 million cases of trichiasis (46.6% of all cases) in Africa, and it is thought to be endemic in 33 of Africa’s 56 countries [3]. Ethiopia is one of five countries that account for 49% of the global active trachoma burden. According to one nationwide survey on blindness, low vision, and trachoma, the national prevalence of active trachoma (either TF or TI) among children aged 1 to 9 years was 26.9% [4]. According to data, Oromia is the region in Ethiopia with the highest prevalence of active trachoma, next to the Amhara region [5].

Trachoma is the leading infectious cause of blindness worldwide. It has been linked to poverty, limited access to healthcare, and a lack of water. Recent data from population-based surveys were provided for 42 of the 57 endemic countries. Active trachoma is estimated to affect 40.6 million people [1]. Active trachoma primarily affects women and children, whereas cicatricial trachoma affects women three times more than men, most likely due to prolonged contact with infected children [6]. Trachoma has a significant impact on the economies of endemic communities, where it is estimated that $2.9 to $8 billion in productivity is lost each year as a result of the disease [7].

The most recent retrospective analysis of national survey data from 38 trachoma-endemic countries found that overall TF1-9 prevalence decreased significantly across the continent, particularly in western and southern Africa, when compared to baseline. However, the decrease in TF1-9 prevalence had been most pronounced in western and southern Africa, with significant active trachoma still present in eastern Africa, particularly Ethiopia [8].

Several interventions are required to prevent trachoma-related blindness. The WHO and its partners support the SAFE strategy for active trachoma control, which includes surgery for advanced disease, antibiotics to clear C. trachomatis infection, facial cleanliness, and environmental improvements to reduce transmission [9–11]. SAFE components A, F, and E are recommended for entire districts (typical populations of 100 000–250 000) where the prevalence of the active trachoma sign, TF, is 5% in children aged 1–9 years. In such areas, all residents should be offered antibiotic treatment on an annual basis, with the number of rounds determined by the most recent estimate of TF prevalence [9, 12, 13].

Despite WHO’s implementation of the SAFE strategy to eradicate trachoma, active trachoma remains a significant public health concern in Ethiopia [7, 18]. It is critical to identify the magnitude and specific risk factors before developing effective and tailored interventions. Reliable prevalence data at the district level are required to plan interventions at the outset, assess their impact, and demonstrate the achievement of elimination goals. Previous small-scale research in Ethiopia has shown that the magnitude and contributing factors of active trachoma in children vary by region. However, the prevalence of active trachoma among children aged 1 to 9 in rural areas, including the research sites, was not well addressed. As a result, the purpose of this study was to determine the distribution of Active Trachoma and associated Risk factors in children aged 1 to 9 years in the Households of Sasiga rural district, Western Ethiopia. This study will help researchers better understand risk factors and how important health interventions are in eliminating active trachoma in the community. It is also hoped that the findings of this study will help shape how health interventions are designed and implemented in rural communities.

## Methods and materials

### Study setting, study design, and study period

A community-based cross-sectional study was conducted among 1–9-year-old children in Sasiga district, East Wallaga Zone, Western Ethiopia, from March to May 2022. The district is 345 km west of Addis Ababa. The projected total population of the district is 117,754 in 2022 [13]. There are 29,439 children aged 1 to 9 years in this total population. There are 27 rural and 5 urban Kebeles in the district. Concerning the health infrastructure, there are six government health centers, 33 health posts, and 24 private health facilities.

### Population

All children aged 1 to 9 who were living in households in Sasiga rural district were the source population, while children aged 1 to 9 who were living in households in randomly selected kebele in Sasiga rural district during the study period were the study population.

### Inclusion and exclusion criteria

All the children belonging to the 1–9-year-old age range who had been living in the district for at least 6 months were included, while all children from 1 to 9 years of age who were unable to undergo a physical examination due to a serious medical illness (other than an eye condition) were excluded from the study.

### Sample size determination and sampling procedures

The sample size for the first objective was determined using the single population proportion formula with the following assumptions; 95% confidence interval, 0.05 margin of error, prevalence of active trachoma (18.4%) [7], design effect of 1.5, and 10% non-response rate. The sample size for the associated factors was calculated using the double population proportion formula with assumptions of 95% CI, 80% power, and a 1:1 exposed to non-exposed ratio. Then, the one that gave the largest sample size was considered. Accordingly, 577 households were enrolled in the study.

A multistage sampling technique was used to select the study participants. In the first stage, 8 Kebeles were selected from a total of 27 rural Kebeles by lottery method. The calculated sample size was distributed to each randomly selected Kebele proportional to the total number of households with 1–9 years of children in each Kebele. Households who had 1–9 years of children were identified and coded. Finally, a systematic sampling technique was used to select HH from each selected Kebele. In case households had more than one eligible child, one child by lottery method.

### Data collection tools and procedures

Data were collected by using a pretested interviewer-administered structured questionnaire that was adapted by reviewing different literature [14–17]. Data were collected by integrated eye care workers (IECW) who have been trained in the management and diagnosis of the eye and were assigned to perform an eye examination. Data collectors were certified, and their certification was obtained no more than 6 months before data collection. The IECWs are tropical data-certified trachoma graders. Each was examined separately by using binocular examination loupes (2.5). The examination of the eye was done by careful inspection of the eyelashes, cornea, limbus, eversion of the upper lid, and inspection of the tarsal conjunctiva. The reporting of eye examination results was based on the WHO grading system [18].

### Dependent variable

Active trachoma.

### Independent variables

**Socio-demographic and economic-related factors include** the age of the head of the household, sex of the head of the household, family size, educational status of the head of the household, occupational status of the head of the household, and income of the head of the household.

#### Child-related factors

facial cleanliness, ocular discharge, nasal discharge, frequency of face washing, lack of hand washing after toileting, and soap used for face and hand washing.

#### Environmental and household-related factors include

water source, distance traveled to get water, absence of latrines, presence of flies, presence of feces, garbage (improper waste disposal methods), and number of rooms.

### Operational definitions

#### Active trachoma

The presence of at least one of the two signs of active trachoma according to the WHO simplified trachoma grading scheme (TF or TI) in at least one eye [18].

#### Adequate water

It is recommended a minimum average of 20 L (1–2 pots) per person per day of water supply for all basic needs was considered adequate.

#### Clean face

A face of the child that was free of eye discharges, nose discharges, or flies at the time of eye examination.

#### Corneal opacity (CO)

the presence of easily visible corneal opacity over the pupil [18].

#### Fly in a home

When there is/is a countable fly in a house during data collection, despite the number of flies.

#### Household

It consists of one or more people who live in the same house.

#### Trachomatis inflammation—follicular (TF)

The presence of five or more follicles each having a diameter of at least 0.5 mm in the central part of the upper tarsal conjunctiva [5].

#### Trachomatis inflammation—intense (TI)

A pronounced inflammatory thickening of the upper tarsal conjunctiva that obscures more than half of the normal deep tarsal blood vessels [5].

#### Trachomatis scarring (TS)

The presence of easily visible scarring in the upper tarsal conjunctiva [18].

#### Trachomatis trichiasis (TT)

The presence of at least one eyelash rub on the eyeball or evidence of removal of in-turned eyelashes in the two weeks before the examination [5].

### Data quality control

The questionnaire was initially prepared in English and then translated into Afan Oromo and Amharic by the language experts, and translated back into English to check its consistency. A pretest was conducted on 5% of the sample before actual data collection in an adjacent kebele, which was out of the study area. Clinical eye examination was performed by integrated eye care workers (IECW) who have been trained in the management and diagnosis of the eye. The reporting of eye examination results was based on the WHO grading system. During the data collection, data collectors were intensively supervised at each site and the completeness and accuracy of data was checked at the end of each day.

### Data processing and analysis

The collected data was checked for completeness, coded entered into EpiData version 3.1, and analyzed by SPSS version 24. Descriptive statistics were computed to describe socio-demographic characteristics and environmental, and child-related conditions. Both bivariable and multivariable logistic regression analyses were performed. Variables with a *p*-value < 0.25 in bivariable logistic regression were entered into multivariable logistic regression. Adjusted odds ratio with 95% confidence interval was used to identify factors associated with active trachoma after controlling potential confounders. Variables having *P*-value < 0.05 in multivariable logistic regression were considered statistically significant factors associated with active trachoma.

## Results

### Sociodemographic characteristics

Five hundred seventy-seven (577) children were enrolled in this study, giving a response rate of 100%. The mean age of the children was 4.59 (± 1.82SD) years, and more than half (58.6%) were females. The majority of household heads were married (82.8%), farmers (64.8%), and could not read and write (38.1%). Slightly more than half (58.1%) of households have less than five family size. Concerning religion and ethnicity of household, most household heads are Promo (89.9%) and protestant religion followers (40.9%) followed by Muslims (31.5%). Almost half 283(49.0%) of the households get an average monthly income above 3000 ETB. Table 1.

**Table 1:** Socio-demographic characteristics of the study participants, Sasiga rural district, East Wallaga Zone, Oromia, 2022 (*N* = 577)

Variables and their categories		Frequency and percentage
Sex of the head of the household	Male	287(49.7%)
	Female	290(50.3%)
Religion of the head of the household	Orthodox	148(25.6%)
	Protestant	236(40.9%)
	Muslim	182(31.5%)
	Wakefata	11(1.9%)
Marital status of the head of the household	Married	478(82.8%)
	Single	15(2.6%)
	Separated	62(10.7%)
	Widowed	22(3.8%)
Ethnicity of the head of the household	Oromo	519(89.9%)
	Amhara	35(6.1%)
	Others*	23(4.0%)
Occupation of the head of the household	Farmer	374(64.8%)
	Daily laborer	24(4.2%)
	Merchant	110(19.1%)
	Housewife	51(8.8%)
	Government employee	18(3.1%)
Educational status of the head of the household	Cannot read and write	220(38.1%)
	Only read and write	182(31.5%)
	Primary education	28(4.9%)
	Secondary and above	147(25.5%)
Monthly income	< 1500 ETB	106(18.4%)
	1500–3000 ETB	188(32.6%)
	> 3000 ETB	283(49.0%)
Family size	< 5	335(58.1%)
	≥ 5	242(41.9%)
Child age	1-4years	307(53.2%)
	5-9years	270(46.8%)
Sex of the child	Male	239(41.4%)
	Female	338(58.6%)

Others*: Gurage, Tigre

### Environmental and household conditions

Near to two-thirds, 372(64.5%) of the households selected for this study were made up of thin and thatched roofs and 535(92.7%) had two or more rooms for living. Most of the households 474(82.1%) had a separate cooking room and only 61(12.7%) of the cooking rooms had windows. Concerning fly densities in the compound of the households, 33(5.7%) of the household’s compounds have quieted 109(18.9%), and 202(35.0%) few densities of flies around their compounds. Near to one-third 179(31.0%), and almost one-fourth 150(26.0%) of the households get water from protected springs and rivers for domestic use respectively. More than half 308(53.4%) of the households used two to three jerrycans (41–60 L) per day and 272(47.1%) of the households traveled an average distance of sixteen to 30 min from their households to water source. Regarding the waste disposal system, 132(46.8%) of the households dispose of domestically produced refuse near their living house. Near two-thirds, 350(60.7%) of the households have a latrine, 305(87.1%) of the households use a latrine, and 163(46.6%) of the latrines are uncovered traditional pit latrines. Of most of the households, 433(75.0%) have cattle and 239(55.2%) of them have separate rooms for their cattle. One hundred seventy-five (30.3%) of the households had feces in their compound. Table 2.

**Table 2:** Environmental and housing conditions of households of Sasiga rural district, East Wallaga Zone, Oromia, 2022 (*N* = 577)

Variables and their categories		Frequency and percentage
Condition of the household roof	thin and clean	62(10.7%)
	grass and clean	107(18.5%)
	thin and thatched	372(64.5%)
	grass and thatched	36(6.2%)
Number of room categories	1.0	42(7.3%)
	>=2.00	535(92.7%)
Have a separate cooking room	Yes	474(82.1%)
	No	103(17.9%)
The cooking room has a window	Yes	61(12.7%)
	No	413(87.3%)
Fly densities in and around the compound	Very few	176(30.5%)
	Few [6–10]	202(35.0%)
	medium in number [11–15]	57(9.9%)
	many in number [16–20]	109(18.9%)
	quite many (> 20)	33(5.7%)
The main source of water for domestic use	River	150(26.0%)
	Unprotected spring	74(12.8%)
	Pond	8(1.4%)
	Unprotected well	61(10.6%)
	Rainwater	25(4.3%)
	Protected spring	179(31.0%)
	Protected well	32(5.5%)
	Pipe	48(8.3%)
Amount of water family members consume per day	Less than 20 L	4(0.7%)
	20 to 40 L	65(11.3%)
	41 L to 60 L	308(53.4%)
	61 L to 80 L	94(16.3%)
	Above 80 L	106(18.4%)
Distance travel to get water mainly for domestic use	≤ 15 min	179(31.0%)
	16–30 min	272(47.1%)
	> 30 min	126(21.8%)
What do you do to your domestically produced refuse	Burn	160(27.7%)
	Bury	62(10.7%)
	Dispose in the farm	74(12.8%)
	Dispose in other places	281(48.7%)
Where do you dispose of it	Nearby living house	132(46.8%)
	Far away from the living house	131(46.5%)
	In the river or stream	19(6.7%)
Do you have a latrine?	Yes	350(60.7%)
	No	227(39.3%)
Type of latrine do you have	Uncovered traditional pit	163(46.6%)
	Ventilated pit latrine	9(2.6%)
	Covered traditional pit latrine	172(49.1%)
	Others	6(1.7%)
Do you use a latrine?	Yes	305(87.1%)
	No	45(12.9%)
Do you have cattle?	Yes	433(75.0%)
	No	144(25.0%)
Separate rooms for cattle	Yes	239(55.2%)
	No	194(44.8%)
Feces present in the compound of the household	Yes	175(30.3%)
	No	402(69.7%)
Took health education about trachoma	Yes	293(50.8%)
	No	284(49.2%)

### Child related conditions

Only one-fourth, 87(24.9%) of children wash their hands with soap after using a latrine, and 275(47.7%) wash their face two to three times per day. During facial observation, 68(11.8%) of children had nasal discharge, 25(4.3%) had ocular discharge, 30(5.2%) had both nasal and ocular discharge, and 35(6.1%) had flies on their face. From a total of 577 observed children, 64(11.1%) children had eye problems and eye discharge was the most commonly found eye problem 40(62.5%) followed by itching 8(12.5%) and redness of eyes 6(9.4%). Table 3.

**Table 3:** Conditions of 1–9 years children in Sasiga rural district, East Wallaga Zone, Oromia, 2022 (*N* = 577)

Variables and their categories		Frequency and percentage
Children wash their hands after the use of the latrine	Yes	87(24.9%)
	No	263(75.1%)
How often does the selected child wash his/her face?	Once per day	156(27.0%)
	Two to three times per day	275(47.7%)
	2 to 6 per week	137(23.7%)
	Once per week	9(1.6%)
Selected children use soap when washing his/her face	Yes No	130(22.5%) 447(77.5%)
Case of the child during facial observation	Normal looking	407(70.5%)
	Ocular discharge	25(4.3%)
	Nasal discharge	68(11.8%)
	Ocular and nasal discharge	30(5.2%)
	Flies on the face	35(6.1%)
	Ocular, nasal discharge and flies on the face	12(2.1%)
The child has an eye problem	Yes	64(11.1%)
	No	513(88.9%)
What is the case of the child	Discharge	40(62.5%)
	Itching	8(12.5%)
	Excessive tearing	5(7.8%)
	Redness of eyes	6(9.4%)
	Photophobia	3(4.7%)
	Swelling of eyelids	2(3.1%)

### Factors associated with active trachoma among 1–9 years children

In logistic regression analysis, variables having a *P*-value of less than 0.25 were selected for multivariable logistic regression analysis. These variables were the sex of the household’s head, educational status of the head of households, monthly income, distance traveled to get water, the number of rooms, the child’s use of soap when washing his/her face, having health education about trachoma, presence of latrine, condition of the house roof, and presence of feces present in the compound of the household. In multivariable logistic regression after controlling potential confounders, the monthly income of the households, distance traveled to get water from water sources, number of rooms, and presence of feces in the compound of households are significantly associated factors at a *P* value less than 0.05. The study found that having low monthly income less than 1500 ETB (*P*-value 0.007, AOR with 95%CI 3.49(1.39, 8.75)), increased distance travel to get water from water sources (greater than half-hour travel)(*P*-value, 0.005, AOR with 95%CI, 8.34(1.89, 36.73)), number of room (households having only one room), (*P*-value 0.045, AOR with 95%CI, 2.98(1.027, 8.68)), and presence of feces in the compound of the households, (*P*- value, 0.005, AOR with 95%CI, 3.08(1.41, 6.75)), were significantly associated with active trachoma among 1–9 years children. Table 4.

**Table 4:** Factors associated with active trachoma among 1-9years children in Sasiga Rural District, East Wallaga Zone, Oromia, 2022 (*N* = 577) Bivariable and multivariable logistic regression analysis

Variables	Category	Active trachoma		COR with 95% CI	AOR with 95% CI
		No	Yes		
		n (%)	n (%)		
Sex of the head of the household	Male	266(51.0%)	21(38.2%)	Ref	1
	Female	256(49.0%)	34(61.8%)	1.68(0.95,2.98)	1.44 (0.67, 3.07)
Educational status of the household head	can’t read and write	191(36.6%)	29(52.7%)	3.57(1.44, 8.82)	1.12(0.31, 4.01)
	only read and write	163(31.2%)	19(34.5%)	2.74(1.06, 7.05)	0.69(0.18, 2.65)
	Primary	27(5.2%)	1(1.8%)	0.87(0.10, 7.52)	2.03(0.17, 23.91)
	Secondary and above	141(27.0%)	6(10.9%)	Ref	1
Monthly income (in ETB)	> 3000	273(52.3%)	10(18.2%)	Ref	1
	1500–3000	91(17.4%)	15(27.3%)	4.500(1.95, 10.37)	1.39(0.48, 4.04)
	< 1500	158(30.3%)	30(54.5%)	5.18(2.47, 10.88)	3.49(1.39, 8.75) *
The distance traveled to obtain water	≤ 15 min	176(33.7%)	3(5.5%)	Ref	1
	16–30 min	248(47.5%)	24(43.6%)	5.67(1.68, 19.15)	1.75(0.39, 7.85)
	> 30 min	98(18.8%)	28(50.9%)	16.76(4.97, 56.55)	8.34(1.89, 36.73) *
number of rooms	>=2	32(6.1%)	10(18.2%)	Ref	1
	1	490(93.9%)	45(81.8%)	0.29(0.136, 0.637)	2.98(1.027, 8.68) *
Children use soap when washing his/her face	Yes	124(23.8%)	6(10.9%)	Ref	1
	No	398(76.2%)	49(89.1%)	2.54(1.065, 6.08)	1.88(0.45, 7.85)
Received trachoma education	Yes	274(52.5%)	19(34.5%)	Ref	1
	No	248(47.5%)	36(65.5%)	2.093(1.17, 3.74)	0.83(0.36, 1.91)
Have a latrine	Yes	328(62.8%)	22(40.0%)	Ref	1
	No	194(37.2%)	33(60.0%)	2.54(1.44,4.47)	1.94(0.83, 4.50)
Condition of the house roof	Thin and clean	55(10.5%)	7(12.7%)	Ref	1
	Grass and clean	80(15.3%)	27(49.1%)	2.65(1.08,6.52)	1.19(0.36, 3.95)
	Thin and thatched	360(69.0%)	12(21.8%)	0.26(0.099,0.69)	0.35(0.10, 1.23)
	Grass and thatched	27(5.2%)	9(16.4%)	2.62(0.88, 7.79)	3.88(0.82, 18.45)
Feces in the compound of the household	Yes	152(29.1%)	23(41.8%)	1.75(0.99,3.088)	3.08(1.41, 6.75) *
	No	370(70.9%)	32(58.2%)	Ref	1

*P-value is significant at 0.05 in multivariable logistic regression analysis

## Discussion

The study found that 9.5% of 1-9-year-old children in the Households of Sasiga rural district had active trachoma. This result is lower than the findings of a study conducted to determine the national prevalence of active trachoma for children aged 1 to 9 years in Ethiopia, which was 40.14% with regional variations; the highest prevalence was found in Amhara (62.6%), Oromia (41.3%), SNNP (33.2%), Tigray (26.5%), Somali(22.6%), and Gambella (19.1%) (26.5%) [5].

The results are also lower when compared to studies conducted in Baso Liben district, Est Gojam (24.1%) [19], Andebet district, North West Ethiopia(35.37%) [20], Dembia district, Northwest Ethiopia (18%) [21], Gazegibela district, Wagehemra Zone, Amhara region (52.4%) [22], Arbaminch health and demographic surveillance site (17.8%) [23], Gamo Gofa Zone, Southern Ethiopia (36.7%) [17, 24], Lare district, Southwest Ethiopia (21.60%) [16], active trachoma and community use of sanitation, Ethiopia (29%) [25], and Baso Liben District of East Gojjam, Ethiopia (24.1%) [14]. The difference might be associated with the impact of SAFE strategy intervention over time. Currently, the Ethiopian Federal Ministry of Health and other stakeholders have focused on scaling up all components of the WHO-endorsed SAFE strategy (surgery, antibiotics, facial cleanliness, and environmental improvement) in the study setting through integrated eye care workers at the primary health care level. The differences also might be related to differences in the study setting, sample size, and infrastructure-related factors in health care.

In this study, trachomatous inflammation-follicular (TF) was found to be the most prevalent type of active trachoma among 1–9-year-old children, accounting for 51 (8.8%) of the total of 55 (9.5%) cases of active trachoma identified. Several studies conducted in various parts of the country back up this finding [2, 16, 17, 19, 21, 23–27].

According to this study, children from lower-income households were more likely to have active trachoma than children from higher-income households. The findings are consistent with those of a similar study conducted among rural 1-year-old children in the Wadla district of northern Ethiopia [2] and the unmatched case-control study conducted in the Rural Area of Gozamn District, Northwestern Ethiopia [28]. The effects of poverty on health care, a lack of hygiene, a high likelihood of sharing tools, a low immunity status, and a lack of information are all possible explanations [2, 29].

*In this study*,* the time it took to get water for home use was found to be highly associated with the development of clinically active trachoma among 1-9years aged children in the study area.* The findings are similar to those of a study conducted among 1-9-year-old children in Arba Minch, southern Ethiopia, in which the odds of developing trachoma among 1-9-year-old children from households that obtain water for household consumption from a distance greater than thirty minutes on a walk away from their homes were nearly three times higher than those from households that obtained water from a distance less than or equal to thirty minutes on a walk away from their homes [23]. The finding is also consistent with research from Tanzania [3, 30] and Ethiopia [14, 21, 31]. According to these studies, as reported water collection times increased, the prevalence of active trachoma in children increased significantly.

## Conclusions

The household distribution of Active Trachoma in children aged 1 to 9 in the study area was high and remains a major public health concern. Monthly income, distance traveled to get water from a source, the presence of feces in household compounds, and an inadequate waste disposal system were all factors associated with active trachoma in the study area. As a result, in collaboration with other concerned bodies such as municipalities and water resource offices, the Regional Health Bureau, Zonal Health Department, District Health Office, and other concerned bodies should work to provide adequate, safe, and accessible water sources, as well as to improve environmental conditions by educating people and facilitating waste disposal systems for households. Furthermore, the district should strengthen SAFE (Surgery, Antibiotics, Facial Cleanliness, and Environmental Hygiene) services and a continuous mass antibiotic treatment strategy supported by trachoma transmission and prevention sanitary education.

## Data Availability

All relevant data are included in this manuscript.

## Abbreviations

AOR: Adjusted Odds Ratio
C. trachomatis: Chlamydia Trachomatis
CO: Corneal Opacity
COR: Cruds Odds Ratio
ETB: Ethiopian Birr
HH: Household
IECW: Integrated Eye Care Workers
PPE: Personal Protective Equipment
SAFE: Surgery Antibiotics Facial Cleanness Environmental improvements
TEO: Tetracycline Eye Ointment
TF: Trachomatis, Inflammation-Follicular
TI: Trachomatis Inflammation-Intense
TS: Trachomatis Scarring
TT: Trachomatis Scarring
WHO: World Health Organization
